# Why is hospice care important? An exploration of its benefits for patients with terminal cancer

**DOI:** 10.1186/s12904-021-00757-8

**Published:** 2021-05-17

**Authors:** Maria Wajid, Eslavath Rajkumar, J. Romate, Allen Joshua George, R. Lakshmi, Srinagesh Simha

**Affiliations:** 1 Department of Psychology, Central University of Karnataka, Gulbarga, India; 2 Head of the Department, Department of Psychology, Central University of Karnataka, Gulbarga, India; 3 Karunashraya, Bangalore, India

**Keywords:** Terminal cancer patients, Hospice, Palliative care need, India

## Abstract

**Background:**

Palliative care has proven over time that it can help prolong life spans with the use of hospices. The literature reports that most patients with cancer are diagnosed in the later stages and since there is no cure, they will require palliative care at some point during their illness. However, most developing countries, including India, have failed to establish hospices; as a result, large numbers of cancer patients are still deprived of palliative care. To initiate better access to hospices, it is important to understand the benefits of the same. Therefore, the present study aims to explore the advantages of hospice care from the perspectives of advanced cancer patients living in hospice centres.

**Methods:**

The present study uses the method of exploratory research. Using purposive sampling, 8 participants living in a hospice in Bengaluru, India were selected and semi-structured interviews were conducted to collect data from the patients. This data was analysed using thematic analysis. Any underlying patterns were determined to identify the evident themes that emerged from the data.

**Results:**

After thematic analysis, 4 themes were identified, namely, pain management, altruism, a good death, and overall satisfaction. Within some themes, there were specific sub-themes that have been illustrated using direct quotes from the interviews.

**Conclusion:**

The findings of this study suggest that hospice centres play an important role in helping patients to come out of the trauma during the advanced stages of cancer. A sense of relief can be given to the patient by implementing palliative care. This is possible by building more hospice centres in the country where all individuals, irrespective of their financial status, can opt for the service. Having palliative care will provide dignified death to the patients.

## Introduction

Cancer is one of the world’s deadliest diseases. Its impact on society is immense and for most people, there is no scarier diagnosis than that of cancer. According to the new estimates on global cancer burden released by International Agency for Research on Cancer (IARC) indicated that there were 19.3 million cases of cancer in the world, out of which 10 million deaths occurred in 2020. The estimates are expected to rise more by 2040 at 30.2 million cases. India alone has contributed to 1.32 million cases and 8,520,000 deaths, India is estimated to contribute 2.09 million cases by 2040 and estimated deaths to occur by 2040 is 1.38 million [10].

As patients are diagnosed in the later stages and as there is no cure, they will require palliative care at some point during their illness [9]. Terminal cancer is a stage of the disease where there is no option for curative treatment; no treatment will eliminate or cure cancer at this stage [8]. Approaching death in the right manner is crucial [29] and it can be achieved through hospices, as the feeling of a good death enhances the medical field’s orientation, approach, and results to a large extent by providing care to patients based on the severity of their illness, providing a good quality of life [20].

Hospice care is an approach that involves people from multiple disciplines in providing comprehensive care for patients nearing their end of life. There are certain criteria for patients who can avail hospice services; e.g., patients must be in the terminal phase of the illness with a life expectancy of 6 months or less. In the US, the eligibility for hospice care is based on prognosis of the life expectancy, which is less than 6 months, and for palliative care, the eligibility is based on need; no prognostic restrictions apply. Other countries are synonymous in these two terminologies. The main care focus for patients is symptom management, which improves the quality of the remainder of their life. Palliative care involves not only the patient but also their family members. Other measures are also taken so that the patients can live life comfortably and maintain dignity [2]. Hospice care focuses on reducing intensive care to prolong life. Avoiding expensive hospitalizations and admitting the patient at a hospice could improve life quality drastically towards the end [34].

According to the World Health Organization (WHO) [33], Palliative care is an approach that improves the quality of life of patients and their families facing the problem associated with a life-threatening illness, through the prevention and relief of suffering through early identification and impeccable assessment and treatment of pain and other problems, physical, psychosocial and spiritual. Too et al. [30] reported that patients in palliative care have experienced improvement in feelings, quality of life, and pain management. An estimated 20 million patients can benefit from the care service globally in a single year. An increase of 58% was observed in the countries that are opting to establish palliative care services; 21 countries have been added to the tally since 2006. Africa has seen many benefits from the setup while the US and Canada have recorded high development in this area [17]. In the US, community-based foundations laid the initial stepping stones for establishing palliative care centers, numbers of which have seen an increase in ratio to 110% over the past 30 years. As the demand was high, this service was extended to hospitals; every 2 out of 3 were equipped with palliative care, showing an increase of 138% since 2000. Among the 234 countries it was observed that 136 of them had established hospice-palliative care services, which has seen an addition of 21 countries establishing such services since 2006 [17]. Providing meaning in life for patients, be it by focusing on the positives in the lives they have lived or viewing their approaching death and giving meaning to it, plays an integral and influential role in their treatment.

However, most developing countries, including India, have failed to establish hospices; consequently, a large number of terminal cancer patients are deprived of palliative care in the end stages of their lives [21].

Hospices also support the family and/or caregiver(s) by providing bereavement services [19]. The aim and designation of hospice care is to give thorough, interdisciplinary, and team-based palliative care to patients entering the last months of their life. This is the primary aim of every hospice and it includes a care quotient for the family as well [20]. Studies have proven that patients experience feelings of being cared for, reduced pain, and positivity [30] in hospices. To have better hospice facilities, it is important to understand its benefits. There is insufficient literature regarding this topic. Research in countries that have successfully established hospice care for large audiences are helpful in the development, implementation, and monitoring of palliative care services for terminally ill patients [22]. The present study aims to explore the benefits of hospice care for patients with terminal cancer from the perspectives of patients staying at Karunashraya hospice in Bengaluru.

## Methods

### Study design

This was a qualitative study where data was collected from interviews and thematically analysed. Thematic analysis is an iterative process that starts from raw data and transforms into more meaningful data. This method involves organizing the data, locating codes, identifying, reviewing, defining, and reporting themes and sub-themes [4]. Therefore, the patterns were determined using thematic analysis to pick the evident themes that emerged from the data.

### Participants

Purposive sampling was employed to recruit participants from a hospice providing palliative care to terminal cancer patients in Bengaluru. Out of all the patients in the hospice who were screened, 8 were chosen as eligible candidates for the study, 3 were male and 5 were female (refer Table 1). The reasons for ineligibility were fluctuating health, shyness, and unwillingness to share their experience. The eligibility of the participants were according to the following criteria:

- Aware of the diagnosis and prognosis of their illness
- Able to communicate
- Proficient in English or Kannada
- Aged 18 years and above
- Have been at the hospice for 2 months or more to have experienced the overall services provided there and would be at better understanding to appreciate the help they received at the organisation.

**Table 1:** Socio-Demographic Characteristics of the participants

Pseudonyms Names	Age	Gender	Cancer	Religion
BA	39	Male	Leukemia	Hindu
BG	38	Female	Carcinoma Breast	Christian
TJ	69	Male	Carcinoma Thyroid	Hindu
RC	45	Female	Carcinoma Mouth	Hindu
LK	44	Male	Carcinoma Mouth	Christian
RK	59	Female	Carcinoma Stomach	Hindu
AF	60	Female	Carcinoma Stomach	Islam
CD	64	Female	Carcinoma Stomach	Converted Christian

Patients with suffering from psychological disorders such as schizophrenia, dementia, and/or autism were not considered ineligible. The selected participants were explained about the study in detail and a convenient time was scheduled to conduct the interviews. Demographic information, such as age, gender, and religion, was also collected.

### Data collection tool and procedure

Before initiating the data collection process, a six-month internship was undertaken by the researcher at the hospice. The medical director of the institute trained the researcher in communicative and questioning skills and various other aspects such as how to initiate and communicate with the patients, ways to conduct interviews, and how to handle various situations that may arise during the interview. Training included lectures, role-plays and rehearsals. Meanwhile, the counsellors of the institute screened for participants using purposive sampling. Prior to the interview, written consent was obtained by the investigator and a brief explanation was provided regarding the study and the rights of the participant, i.e., they had the liberty to refuse to answer any question or even withdraw from the study. The participants were reassured about privacy, confidentiality, and the freedom to express their views. Semi-structured interviews were conducted. The interview guide was prepared using a review of the literature and was translated into the local language, Kannada, by a professional translator. The interview guide included questions such as “What are the most important things in your life right now?”, “How has life changed after coming here?”, “What is the best thing about living here?”, “How do you think your death could be made easier?”, “What would you like in your future care?” Phrases such as “Go on”, “Could you explain more?”, “Like?” etc. were used to get further clarification. The counsellor of the institute also attended the interviews to oversee the situation in case the patient felt uncomfortable. The investigator audiotaped and transcribed these interviews. The average duration of interviews was found to be 34 min.

### Data analysis

All 8 interviews were included in the analysis. The investigator transcribed the audio recordings and (in cases where the participant communicated in Kannada) translated them to English. Thematic analysis was used to analyze the data through the following steps:

1. Thoroughly reading the transcripts to grasp all the minute details
2. Constructing codes based on recognized similarities
3. Identifying all potential themes and sub-themes
4. Reviewing the identified themes to ensure that they reflected the dataset
5. Labelling the themes to give precise definition
6. Report was then clearly and convincingly written, explaining the stories based on the data analysis [4].

### Ethical approval

The study was underway after seeking the approval of the ethical committee at Karunashraya. Patient’s health condition was given importance; the interview was discontinued if he/she was unable to continue. Furthermore, ward nurses were asked to maintain close supervision on the interviewed patients and counsellors to conduct a separate session to check on any negative influence on patients after the interview.

### Socio-demographic characteristics

Table 1 shows the socio demographic details of the participants. A total of 8 participants were enrolled for this study. The mean age of the participants is 52.25 and Standard Deviation is 12.09. Regarding religion, 50% were Hindus, 40% were Christians, and 10% were Muslims (Refer Table 1).

## Results

The analysis of the interviews presented 4 main themes:

i. Pain management
ii. Altruism
iii. Good death
iv. Overall satisfaction

These themes demonstrate the challenges that advanced cancer patients experience and have been illustrated using direct quotes from the interviews.

### Theme 1: Pain Management

Pain affected the participants psychologically due to the feeling of loss of control which led to negative behaviours such as suicide attempts. Pain is an important concern for patients as it was intolerable and difficult to manage. BG said,

Because of the intolerable pain, I have tried to commit suicide more than 10 times. The cancerous area had developed maggots that I had to remove myself. I would think that these should have been on my dead body and not on me now. There was nobody to help me during my pain and suffering.

When asked if they felt any difference being at the hospice from home, their answers were satisfying as they mentioned that their pain had diminished and expressed satisfaction for receiving such good care services. BA said, “I suffered a lot in the past but after coming here, I feel better. Now, I don’t feel any pain and all.” The hospice was a boon to each participant in terms of relieving their pain through effective pain management rather than suffering at home.

### Theme 2: Altruism

It is observed that patients acquired feelings of altruism because of the help they receive at the hospice. Sub-themes included kindness to others while living and organ donation.

#### Eagerness to help

Since most patients were treated with love and respect at the hospice, their physical, emotional, and social needs were taken care of. Most participants stated that they also desired to help others. The predominant reason behind this was the help and medical attention they had been receiving from the hospice, likes of which is not affordable for them. BA remarked, “Besides my personal goals, I had always wished to contribute to society. After coming to the hospice and seeing how much they are doing, this wish was reinforced further.” RL said,

I am not a nurse, but if I could return the care provided by Karunashraya, even by doing little things like writing records or work in the office, I would do so without hesitation.”

Moreover, because the patients had endured agony and depression, they wished to be able to alleviate these feelings for others. BG expressed,

If God gives me a healthy life, then I want to help orphans and the needy. I have endured a lot of pain in my life. Nobody should experience similar hardships. That’s why we need to help each other.

Palliative care had set an example of benevolence and selflessness that inspired the participants to be of help for and assuage he pain of others. The informants were able to find meaning in life by helping for others. “It is after coming here that I have found purpose in life, which is to help others because life is very short. I believe everybody must be pure at heart and good to each other,” TJ asserted.

This desire to help others is because of the happiness they had gained at the hospice. The compassionate they received provided them with a sense of purpose and meaning and also helped boost self-esteem, which was lost in the course of the illness.

#### Token of gratification

A few participants had expressed their desire to donate their organs after their death as an expression of gratitude. Participants felt that they have a duty toward their fellow human beings by helping them any way they can because of all the help they have been receiving at the hospice. They felt that by doing so, they might save lives and simultaneously improve many lives related to the recipient. BG said,

If any part of my body is good enough for people to use it, be it the eyes, ears, or any part, I would like to donate. I don’t want others to suffer like me. I have informed my family that my decision is to be respected and obliged.

BA asserted,

If organ donation is useful, then why shouldn’t we do it? I feel that it is noble work. After coming here, I definitely want to donate my organs because they help us so much, and the least I can do in return is help others by organ donation.

One informant expressed that the organs be taken only if they are deemed good for a normal human being because they don’t want to be the reason for pain in others. Such an act of kindness will provide them a sense of respect and fulfilment in their last days.

### Theme 3: Good Death

Thoughts regarding dying a good death endeavoured among the participants. They expressed their beliefs on how they wished to die. Sub-themes include painless deaths, being in a good place, and not disturbing others.

#### Painless death

Most participants expressed the wish to die without any pain, maintaining that death should be quick and painless. BA said,

Death should come to me without pain. *Cries*. I want to die without knowing it, just close my eyes and without any suffering. I wish it would happen when I am least expecting it, maybe while I am just sitting.

Informants were also observed being considerate of others’ feelings; a death that does not cause others distress was also considered a good death. BG also mentioned, “I want to die with absolutely no pain.”

The desire to die without pain was the primary wish that patients expressed when asked about their views on a good death. They felt they had already endured much and wished for a painless death.

#### Good place

According to BA, the place of death was an important factor for a good death. The value of the place in which they want to die symbolizes the connection with the place. It is seen that certain feelings associated with the place bring a sense of comfort at the time of death. An individual might have led a lifetime at a place where peace and tranquillity was found, like the house they have lived in. BA stated, “A good place is also important for me to die. A peaceful place like Karunashraya is preferable.”

As the participant was suffering from the illness at home before coming to the hospice, they associated discomfort and pain with their home, which was obvious and noticeable. Consequently, they wished to die at the hospice instead of at home because of the feeling of security and comfort that they had experienced at Karunashraya. They felt that the hospice would take good care of them by making them feel at ease during the end of life.

#### Consideration of others’ distress

Participants had seen themselves face the odds, enduring stress, pain and anxiety all the while. These unending worries due to the cancer also resulted in the emergence of the feeling of being a burden to the caregiver, their family and people around them. The perception of themselves as a burden arose with the need to die without troubling others because by now, the informants felt that they were a physical and emotional burden for their near and dear ones. BG asserted,

The person next to me shouldn’t feel annoyed by me. They shouldn’t think, “Ayyo, why is she crying so much?” or “Why is she cribbing so much? She disturbs me sleep at nights with her whining”. No one should ever feel like this about me. I shouldn’t be a burden to others.

RL says,

I am a person who never likes to hurt anybody because I have gone through a lot since I was a child and I know how people feel when they are disturbed or hurt. So, I don’t want to be a burden on others or trouble them.

The informants were aware that the illness had reached a stage where they might have to die in the near future. Maintaining self-sufficiency for as long as possible was their goal besides dying without giving people around them a chance to grumble about them being a burden or even being a reason for bother. They wished to be remembered by others on a good note. The perception of a good death of the informants can be made a possibility in hospices.

### Theme 4: Overall Satisfaction

Sub-themes include a feeling of contentment and family patronage.

#### Feeling of contentment

The participants expressed their gratitude for their care in the hospice. They expressed their happiness and satisfaction after coming to the hospice because their needs were being fulfilled with care, support, and compassion. They were content and did not wish for more than what was being provided. BA said, “I feel better, sir, and in fact, it’s very good here. From the treatment to the service, everyone, including the doctor, counsellor, and nurses, support me so much. I feel satisfied and happy.” LK stated, “After coming here, I’m getting rest and get dressed on time. I feel good here, ma’am.”

BG mentioned,

They look after me so well here. I have a feeling comparable to being at *vaishnava vaikunta* (god’s house). I cannot stop myself from saying repeatedly that people here have taken care of me so well. Even my children and my mother didn’t take care of me so well. Sometimes, when I am feeling sad, nurses here try to console me and the doctors also give me hope. My life is totally different and I am happy living here.

RL said,

I became content after coming here. This place feels like home, more than my actual home does. They are very warm and caring here. I have never seen such a good place, clean too! They don’t hurl it onto the table; they give it with love and affection. You actually feel like eating!

The hospice attended to the participants’ every worry, causing the participants to feel content and comfortable. Participants likened the hospice to their own home and even to God’s home because of the level of care that they received.

#### Family patronage

The participants expressed that it was difficult for their family members to manage them because of emotional and mental stress and physical disturbance due to their illness. The informants were dependent on their caregivers for physical assistance, which was seen as a matter of concern among most patients. While the participants observed the amount of attention they needed, mental disturbance among the family members had developed as a result. BG said,

Nobody has the strength to take care of me at home, madam. Mother can’t as she is old. My elder sister stays with her husband. I can’t ask anybody. I don’t want to trouble anyone either.

RL mentioned,

If at all anything has to happen or if I feel I am being a burden on my family, I will just walk away and go to Karunashraya. I will hand over all the things there. I will stay there till I die because I know they will give me love and affection.

The informants understood the additional distress they caused to their family members and caregivers. However, participants expressed that the hospice provided support to the family during the time of crisis. It stood as a pillar of strength.

The researcher also observed that female had more faith and belief in god whereas religion was not a coping mechanism for males.

## Discussion

Participants in this study mentioned experiencing physical pain associated with their terminal illness and its effects on their mental health. Rumsey et al. [27] reported that 60 to 90% of advanced cancer patients experience excruciating pain. This leads to many issues such as discomfort, stress, frustration, and anxiety. Pain relief and palliative care are integral to cancer care. When hospice patients who are in their pain are in control, it results in a better life quality, relationships and emotional support and helps the family members to feel less burdened. Researchers have stated the suffering of the patients was intense at the end of life and pain was the major concern. This then leads to other feelings such as exhaustion, depression, psychological distress, and self-isolation, which directly impact life quality [26]. Patients and their family members feel helpless when they have to take care of the patient at home. It is seen that most caregivers are frustrated, depressed, and helpless. According to research, the caregivers themselves show psychological stress as they tackle the psychological emotions of the patients at the end of life [5]. Studies have shown that about 4 to 70% caregivers went through anxiety, depression, and psychological distress [13, 25].

Hospices could be a source of satisfaction for terminally ill patients. As this study shows, participants, after receiving help and care at the hospice, acquired a sense of altruism at the end stage of life; they wished to become a source of happiness to people around them. This could be the last spring of pride and fulfilment they could achieve. Most participants in this study had a common response to help society by donating their organs after death. Donating organs is like a life-enhancing opportunity for the participants. One participant mentioned that we are all born with a purpose; hence, this could be the purpose that they are fulfilling. Such considerations with terminally ill patients may not stand a chance in reality, but it is positive to note that patients are utmost happy with care and display their token of gratitude to the world and needy.

In this study, it was also found that participants were looking for moments of satisfaction before life ends. Patients might have endured hardships during the course of illness but their stay at the hospice has brought in overall satisfaction in their life. Informants also mention that they were heard empathetically about their illness and worries and care was provided to a level that they were utmost satisfied. Improving the satisfaction quotient also lowers the pessimistic emotions of the patients [3, 18]. Lin & Bauer-Wu [14] reported that the major signs of the quality of life are a positive attitude, relaxed mind, sense of satisfaction and wellbeing. The feeling of satisfying life leads to achieving a good death [6]. Having a good death was also influenced by not being a burden on others and getting to choose the place of death as it gives them a feeling of comfort while embracing death after having found solace in life.

In addition, hospice shares equal importance between the living and dying and this represents the best of palliative care [16]. The Institute of Medicine defines good death as: “decent or good death is one that is: free from avoidable distress and suffering for patients, families, and caregivers; in general accord with patients’ and families’ wishes; and reasonably consistent with clinical, cultural, and ethical standards” [24]. Facilitating good death relieves physical pain along with addressing the psychological and spiritual needs of the patient by reducing the fear of death [31]. Psychologists have a role in the expansion of care given to the terminally ill patients and also the family by helping them achieve a good death. The quality of death is positively correlated with the settling of one’s affairs and timing the death appropriately [32] and the place of death [7].

The initiation of building hospices will not be a monetary burden on the government as palliative care is not high-cost expenditure as compared to other specialties [2]. Yet a large portion of India lacks palliative care facilities except for Kerala [17, 28]. Even in the area that covers palliative care, it is only available in major metropolitan cities, this makes the rural population left out in availing the facility because of lack of awareness, absence of insurance, poverty and high charges on transportation [1, 11, 15, 23]. Out of 908 palliative care services in India, 841 are in the state of Kerala. This shows an imbalance in the availability of services in the country (National Strategies for Palliative Care in India). It has been observed in parts of India where palliative care is established, individuals are benefiting from it. Hence, making hospice available within reach will be of benefit to the patients with terminal cancer to achieve quality of life and good death. These care centres give a unique meaning to the end of life [12].

### Limitations and future directions

While consistent effort was made to conduct this paper with minimum limitations, there still exist a few. This study involved only one hospice in Bengaluru, India. Sample is limited due to the difficulty in gaining consent of patients for reasons such as being unwilling to participate, unaware of their diagnosis and prognosis or other health issues. Future studies are recommended to incorporate larger numbers of participants from different hospices and the views of the caregivers.

## Conclusion

From this study, it is observed that terminal cancer patients have been benefiting from hospice care. It is seen that the selected hospice has been of great help to the patient as it brought about a sense of care and relief. The same can be achieved for higher numbers of patients by establishing more hospice centers that are accessible. Further studies revealed that palliative care service establishments are cost-efficient when compared to other medical facilities. Building such centers will increase the ratio of patients availing these services, provide dignified deaths to patients and give support to their family and caregivers.

## Data Availability

The data will be available from the corresponding author on reasonable request and requirement.
